# Flexible endoscopic evaluation of swallowing in children with type 1 spinal muscular atrophy

**DOI:** 10.1007/s00405-022-07685-0

**Published:** 2022-10-08

**Authors:** Jana Zang, Jessika Johannsen, Jonas Denecke, Deike Weiss, Jana-Christiane Koseki, Almut Nießen, Frank Müller, Julie Cläre Nienstedt, Till Flügel, Christina Pflug

**Affiliations:** 1 Department of Voice, Speech and Hearing Disorders, University Dysphagia Center, University Medical Center Hamburg‐Eppendorf, Martinistrasse 52, 20246 Hamburg, Germany; 2 Department of Pediatrics, University Medical Center Hamburg-Eppendorf, Hamburg, Germany

**Keywords:** Spinal muscular atrophy, Deglutition, Flexible endoscopic evaluation of swallowing, Dysphagia, Onasemnogene abeparvovec, Nusinersen, Risdiplam

## Abstract

**Purpose:**

This study aimed to report on implementing flexible endoscopic evaluation of swallowing (FEES) in infants and toddlers with type 1 spinal muscular atrophy (SMA). In addition, a comparison of FEES results and clinical scores was carried out.

**Methods:**

A prospective pilot study was conducted including ten symptomatic children with SMA type 1 (two *SMN2* copies). They started treatment with one of the three currently approved therapies for SMA at a median age of 3.8 months (range 0.7–8.9). FEES was performed according to a standard protocol using Penetration–Aspiration Scale (PAS) and Murray Secretion Scale as a primary outcome. The Children's Hospital of Philadelphia Infant Test of Neuromuscular Disorders (CHOP-INTEND) for motor function, Neuromuscular Disease Swallowing Status Scale (NdSSS), Oral and Swallowing Abilities Tool (OrSAT), and single clinical swallowing-related parameters were also assessed.

**Results:**

Distinct swallowing disorders were already evident in eight children at inclusion. The most common findings from FEES were pharyngeal secretion pooling, penetration, and aspiration of saliva and food as well as delayed initiation of swallowing. Despite an average increase in motor function, no comparable improvement was found in swallowing function. None of the surveyed clinical scores showed a significant dependence on PAS in a mixed linear model.

**Conclusions:**

Valuable information regarding the status of dysphagia can be gathered endoscopically, particularly concerning secretion management and when oral intake is limited. Currently available clinical tools for children with type 1 may represent a change in nutritional status but are not yet mature enough to conclude swallowing ability. Further development is still required.

## Introduction

Dysphagia is a serious symptom in children with type 1 spinal muscular atrophy (SMA) [1–3] and is likely to affect all children at an early age if left untreated [2–4]. SMA is a rare autosomal recessive neurodegenerative disease affecting approximately one in 10 000 live births [5]. The most common form is the clinically classified SMA type 1, which is usually associated with one to three copies of the *SMN2* (survival motor neuron) gene [6, 7], resulting in a severe course and early onset of symptoms before the age of 6 months. If left untreated, the children can never sit, mostly die within the first 2 years of life, or become dependent on supportive ventilation [8]. Due to the rapid death of motor neurons, complete loss of swallowing function is possible within a few weeks, requiring close monitoring of swallowing ability [1, 2, 9].

Significant progress has recently been made with the launch of SMA newborn screening in Germany in October 2021 and three approved therapies for SMA. Nusinersen is a recurrent and lifelong medication administered by intrathecal injection [10, 11] and acts as a splicing modifier of the *SMN2* gene [12]. Onasemnogene abeparvovec is applied as a single dose intravenous. It works by providing a copy of the *SMN1* gene via an AAV9 vector [13]. Risdiplam is orally administered daily and also modifies the *SMN2* pre-messenger RNA splicing [14].

These new therapies benefit SMA type 1 patients' survival and motor skills [14–19]. Despite the dramatic impact of dysphagia on disease progression, the effects on swallowing function have not yet been adequately considered in the approval studies. Some of these studies recorded the start of tube feeding, adverse respiratory events, or suspected swallowing or feeding problems without further specification. These data cannot determine whether there was pharyngeal dysphagia or a general reduction in food intake. Initial studies that have examined swallowing based on small case numbers caution against unsafe swallowing [3], even in treated cohorts [2].

A retrospective study by Choi and colleagues [3] showed an apparent decrease in the nutritional status in an untreated sample of eleven children with SMA type I. The eight-point Neuromuscular Disease Swallowing Status Scale (NdSSS [20]) was used, an extended oral intake scale developed for progressive neuromuscular disease taking into account secretion management. At the age of 17–24 months, all but one child scored 1 on the scale, which means *tube-fed with oral suction due to no swallowing ability,* and one child scored 3, meaning *tube-fed with occasional oral intake*. Van der Heul et al. [2] reported on parameters from the clinical swallowing examination relevant to swallowing in a prospective cohort of nusinersen treated and untreated children with type 1 SMA. Using wet breathing as an indicator of unsafe swallowing, data were collected at baseline, 2, 6, and 10 months after the start of the therapy. The children from the nusinersen group initially showed an improvement or preservation of their swallowing functions but deteriorated again after 5 months. Berti and colleagues conducted a retrospective analysis of possible clinical markers for oral abilities and feeding in an untreated cohort of 24 children [21]. Recently Weststrate et al. [9] retrospectively assessed the bulbar function of 24 symptomatic children with type 1 SMA at the initiation of nusinersen and 6, 12, and 24 months after initiation. They used the six-point pediatric functional intake scale (p-FOIS) adapted from the original FOIS [22]. Comparable to the results of van der Heul and colleagues, there was neither improvement nor significant preservation of swallowing function despite improvement in motor function.

Only a few studies reported on instrumental swallowing examination and had only a single data collection point. Durkin and colleagues [23] mention a "video swallow study" on nine children without further specification. They report abnormal findings in all children, ranging from delayed swallowing to severe aspiration. Van der Heul et al. [2] conducted a videofluoroscopic swallowing study (VFSS) in five children treated with nusinersen. Silent aspiration was reported in four of five children and laryngeal penetration in the non-aspirating child. Choi and colleagues [3] systematically report results from VFSS in six untreated children. Silent aspiration (penetration–aspiration scale [24], PAS 8) was found in five children; one child showed no penetration or aspiration (PAS 1). Premature bolus loss occurred in one child, laryngeal elevation was impaired in all children, and post-swallow residue was seen in four children.

So far, no data on FEES in this population has been published; however, FEES is a gold standard in dysphagia diagnostics; it does not cause radiation exposure, and, unlike VFSS, it is widely available. Nevertheless, the implementation by experienced diagnosticians in this vulnerable group must be emphasized.

In the present manuscript, we report on the endoscopically collected data concerning the swallowing ability in children with symptomatic, medication-treated type 1 SMA with the following objectives: (i) to report on the implementation of FEES, (ii) to investigate the occurrence and severity of swallowing disorders in this sample, (iii) to investigate the association between clinical scores and FEES-based swallowing outcome, (iv) and to provide a basis for future studies.

## Methods

The local ethics committee gave ethical approval (2020-10329-BO-ff). All parents gave written consent for their child to participate in the study. The sample is from a larger feasibility study of dysphagia in children with rare diseases CHILDYS-RD.

All children with 5q-associated SMA with two copies of SMN2 treated with one of the three currently approved therapies and registration for FEES at a university dysphagia center between August 2020 and February 2022 were prospectively enrolled in this single-center pilot study.

### Clinical examination

Two experienced pediatric physiotherapists assessed motor function using the Children’s Hospital of Philadelphia Infant Test (CHOP-INTEND [25]). The score ranges from 0 to 64, with a higher score indicating better motor development.

The CSE was carried out by an experienced speech-language pathologist (SLP). Non-nutritive sucking (NNS) was tested with the help of a pacifier and scored as *no sucking reflex/unable to suck on pacifier, weak sucking* (not possible to hold the pacifier against a slight resistance), or *sufficiently strong sucking*. Fatigue and coughing during eating, as well as wet breathing, were recorded. In orally fed children, a typical feeding situation was observed.

Parents' reported average number of oral suctions per day was recorded at each measurement point, and where applicable, the initiation of tube feeding or adverse events (e.g., pneumonia) and serious adverse events suspected to be related to dysphagia or poor secretion management (e.g., respiratory failure requiring intensive medical care or resuscitation).

The NdSSS [20] was determined for all subjects at all measuring points by an SLP. The scale ranges from 8 (*totally orally fed with no restrictions*) to 1 (*tube feeding with saliva suction*). It was translated into German by the first author, as there is no comparable valid German-language scale to date. No data on the reliability of the German translation was collected as part of this study. Score 8 was given if infants exclusively drank milk without problems up to 10 months of age, because we found them age-appropriate, *fully orally fed without restrictions*. Choi and colleagues [3] gave this a score of 7 in this case (*the patient eats without something difficult to eat*).

The OrSAT, suggested for assessing oral abilities in children with SMA ages 0–24 months [21], was also translated into German and scored retrospectively. The checklist is based on information provided by the caregivers. It covers aspects of swallowing abilities, including meal duration and fatigue and basic syllable and word production. The possible scoring ranges from 0 to 12 for children aged 10 to 24 months, with a maximum score of 10 for infants aged 6–9 months and a maximum score of 7 for infants younger than 6 months [21].

### FEES protocol

**Fig. 1:**
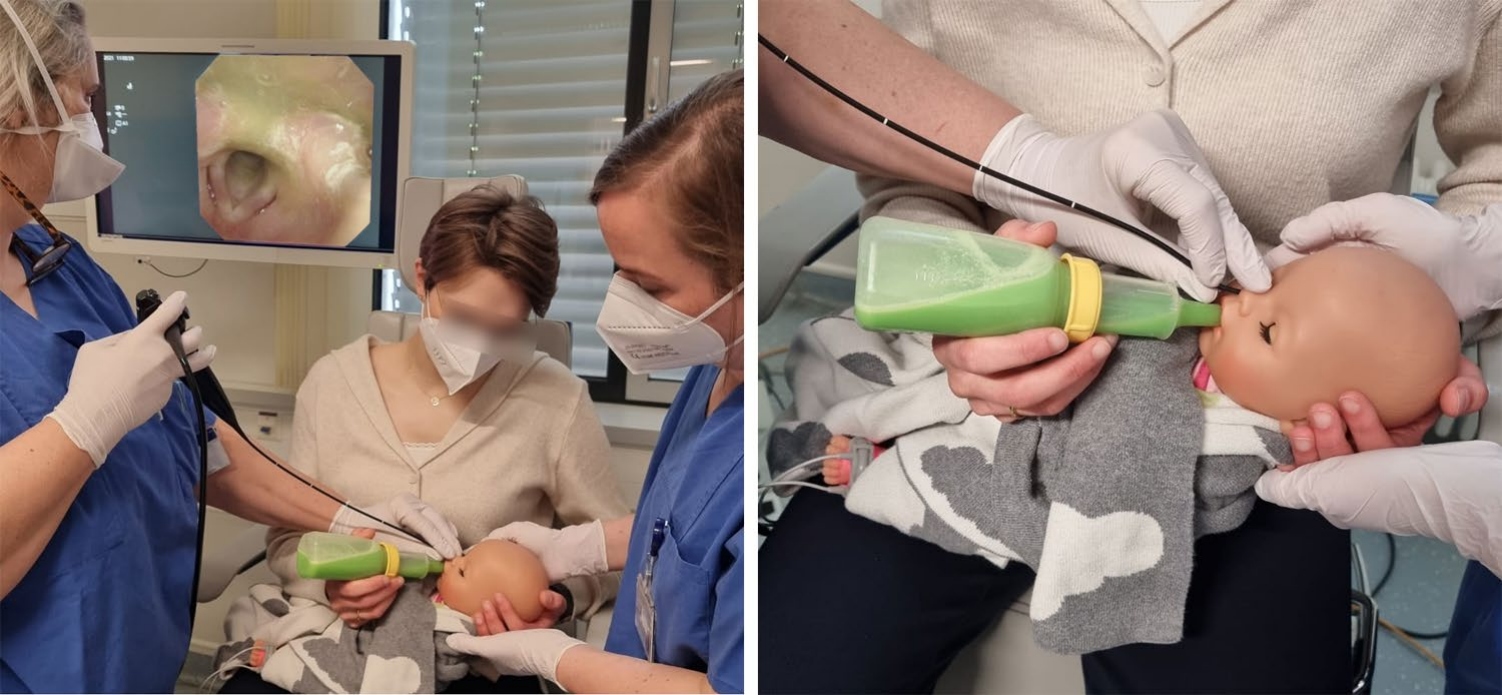
FEES procedure in a semi-reclined position (© Zang, 2022)

FEES was performed as an objective method by experienced specialists in phoniatrics and ENT (> 5 years of experience in pediatric FEES) at a university dysphagia center using a 2.6 mm diameter high-definition rhino-laryngo-videoscope (ENF-V3, Olympus Medical Systems Corp., Japan), and accompanied by an SLP and a nurse. Suction and resuscitation equipment was kept on standby. Oxygen saturation and heart rate monitoring were used in children with chronic respiratory failure. One drop of nasal decongestant (Otriven, Xylometazoline 0.025%) and 0.1 ml of topical viscous lidocaine (via syringe, Xylocaine Gel 2%, Aspen, Germany) were routinely applied. Nasogastric tubes were not removed. Children usually fed in a lying position or whose saturation went down in an upright posture were examined while lying on the caregiver's arms or in the buggy (Fig. 1). After the endoscope was inserted transnasally, breastfed infants were examined in the preferred breastfeeding position.

A pacifier with glucose solution (30%) or interesting toys were used to calm the children. The boluses administered were age- and development-appropriate (mainly milk, water, or puree). All boluses were colored green (“Condi Light Green” E104 Quinoline Yellow + E132 Indigotine I, Schreiber-Essenzen GmbH & Co KG, Germany) and administered via the usual bottle or spoon that the children were used to or with a syringe. The color was diluted about 1:50 to 1:100 in liquids. To approximately 125 g of puree, one ml of dye was added. No color was added in breastfed children. Liquids were thickened with a thickener containing locust bean gum (Aptamil-Antireflux, Nutricia Milupa, Germany) if necessary. The degree of thickening is not specified as a standard in the protocol.

The SMA FEES protocol was developed based on our clinic’s protocol [26, 27] for infants and toddlers according to the standard Langmore protocol [28] and to adaptations suggested by Miller, Schroeder, and Langmore [29].

The procedure starts with *observing anatomical structures* of the nasal airway, nasopharynx, oropharynx, hypopharynx, and larynx, particularly focusing on *secretion management*. Subsequently, the Murray Secretion Scale [30] is scored from 0 to 3 as *normal, pooled bilateral secretions in the valleculae and pyriform sinus, transient secretion in the laryngeal vestibule, and permanently pooled laryngeal or pulmonary secretions*. Changes of positioning or suction are provided to have a detailed picture of secretion pooling and the ability to clear the larynx.

If too little bolus material is transported to the hypopharynx, later on, the eight-point Rosenbek's penetration–aspiration scale (PAS) [24] for secretion is evaluated at this point. The *direct assessment of swallowing* starts with the child's preferred modality and consistency in the case of oral feeding (e.g., breastfeeding, puree). In children who are not orally fed, about 1 ml of slightly thickened water (IDDSI Level 2) is administered with a syringe. Initiation of the swallowing reflex is scored as *normal, delayed* (without specifying a time), or *none* [31, 32], spillage on a scale from 0 (*bolus behind the tongue*) to 4 (*falls into the laryngeal vestibule*) [33]. PAS and Yale Pharyngeal Residue Severity Rating Scale (YPRSRS [34]) are scored for all consistencies. Narrow Band Imaging (NBI) is switched on temporarily to visualize the bolus better. A green-colored bolus illuminates bright red under NBI and enhances the detection of penetration and aspiration in FEES [35]. None of the scales used are validated for children. In case of an unelicited swallowing reflex or inadequate airway protection (PAS > 6), oral suction is performed, and the swallowing assessment with food is stopped. In orally fed children, *compensatory strategies* such as changes in positioning are provided. *Sensory testing* is performed using the touch method as the last point and scored as *intact, weak, or absent* (e.g., Marian et al. [36]).

For *the feasibility and safety* of FEES, the child's behavior (*can be calmed down, screams excessively, refuses to eat*), and if complications occurred (e.g., epistaxis, hypoxia) or the examination had to be stopped were documented. It was agreed that parents would communicate if they felt their child was not doing well.

### Data analysis

Descriptive analysis was conducted for the description of the sample. Further statistical analysis was performed using SPSS Statistic version 27 (IBM, USA). To test the association between clinical scores and FEES, a linear mixed model was calculated with PAS as the dependent variable, CHOP-INTEND, NdSSS, and OrSAT as a covariate, and the case ID as the random intercept. *p* values < 0.05 were considered significant. If the 95% CI for the linear model did not include 0, a significant influence was assumed.

## Results

### Sample

Ten symptomatic children were enrolled in the study, and all parents of these ten children gave written informed consent to participate.

The sample consisted of five girls and five boys with 5q-associated SMA with two *SMN2* copies. The median age at onset of symptoms was 2.0 months (range 0–7.0) (Table 1). Nine children received gene replacement therapy with onasemnogene; five had already been treated with nusinersen in advance. No data were collected prior to therapy with nusinersen. One child received therapy with risdiplam (Table 2).

**Table 1:** Patient characteristics (*N* = 10)

Sex F:M:D	5:5:0
Median age at onset of disease symptoms	2.0 (0.0 –7.0)
Median age at start *SMN* modifying medication	3.8 (0.7 –8.9)
Median age at inclusion	9.4 (0.4 –25.2)
Median CHOP-INTEND score at inclusion	26.5 (13 –55)
Number of children with tongue fasciculation	10
Number of children with adverse events suspected to be related to dysphagia	6
Nutrition at inclusion oral tube feeding (NGT; PEG)	5 5 (4 ;1)
Median age at start tube feeding	6.8 (0.1 –21.9)

All ages in months, median with range (in brackets); D = *diverse*, includes all who do not fit male or female gender

*NGT* nasogastric tube, *PEG* percutaneous endoscopic gastrostomy

**Table 2:** Detailed patient characteristics related to treatment, and adverse events

Case	Symptom onset	Start treatment	Adverse event suspected in relation to dysphagia	Coding	Age at event	Start tube-feeding
1	2.5	3.5 (N); 6.3 (O)	Aspiration pneumonia, ICU (no IV) Pneumonia (viral)	SAE AE	4.2 23.2	4.2
2	1.4	1.6 (N); 10.0 (O)	Bronchopneumonia	AE	17.9	2.2
3	5.0	8.1 (O)	Respiratory failure, ICU (no IV)	SAE	7.5	6.8
4	4.0	5.3 (O)	Pneumonia, respiratory failure, resuscitation, ICU (IV)	SAE	12.6	12.6
5	3.0	4.6 (O)	Parainfluenza Virus, secretion obstruction, resuscitation, ICU ( IV)	SAE	9.7	13.3
6	1.1	5.1 (N); 55.9 (O)	None	–	–	21.9
7	1.1	2.3 (N); 25.2 (O)	Pneumonia (viral)	AE	16.7	–
8	1.4	2.3 (N); 9.7 (O)	None	–	–	–
9	1.0	8.9 (R)	None	–	–	–
10	0.0	0.7 (O)	None	–	–	0.1

All ages in month

*N* nusinersen, *O* onasemnogene, *R* risdiplam, *SAE* serious adverse event, *AE* adverse event, *ICU* intensive care unit, *IV* invasive ventilation

All children received preventive non-invasive ventilation through Bilevel positive airway pressure (BiPAP) at night. One child received permanent mechanical ventilation 6 months after the start of therapy due to a severe adverse event. Five children were already exclusively tube fed at inclusion in the study (Table 1).

Four boys in the sample had at least one adverse event, and all serious adverse events could be assigned to boys. Two out of five girls had an adverse event; three were event-free (Table 2).

### Clinical examination

**Fig. 2:**
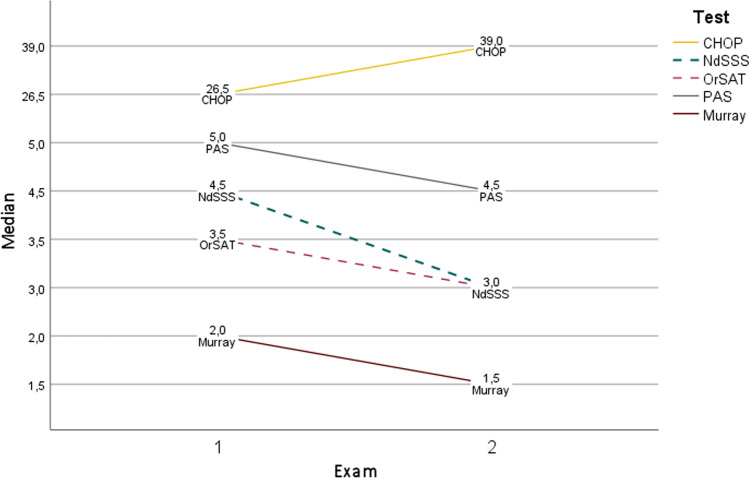
Median CHOP-INTEND (0–64), PAS (8–1) and Murray (3–0) improve, NdSSS (1–8) and Orsat (0–12) deteriorate (dashed lines)

Median values for CHOP-INTEND (motor function), NdSSS, and OrSAT are shown in Fig. 2. The CSE revealed that tongue fasciculations were already present in all children at inclusion. Non-nutritive sucking on a pacifier could not be elicited in one child at inclusion and in another child at follow-up; weak sucking was seen in six cases at inclusion, and in five at follow-up. Sufficiently strong sucking was possible for one child at inclusion, and for two at follow-up. Two children could not be tested, because they refused a pacifier. Fatigue related to feeding was seen in six of nine children at inclusion and in three of eight children at the second examination. This item was not applicable for one child at inclusion and two children at follow-up, because they were never orally fed. The same was true for the item coughing while eating (seven of nine at inclusion and seven of eight at follow-up). All but one child showed a periodically wet breathing sound. Eight children already required oral saliva suction at inclusion. In five of these, suction was necessary several times a day.

### Flexible endoscopic evaluation of swallowing

A total of 18 FEES were carried out with a mean duration of 5 min (SD 2.4, range 1.7–11.1). The median age at first examination was 9.4 months (range 2.7–25.2). About 6 months later, a follow-up was performed, including one high outlier (MDN 5.9, range 2.3–36.8). In one case three FEES were performed, in six cases two FEES, and in three cases one FEES. All children had at least two clinical swallowing examinations.

In all FEES no serious complications occurred. In one child the heart rate increased significantly (195 bpm) at the follow-up examination. It was decided not to offer an oral bolus as it was difficult to soothe. After the examination, it gradually calmed down. The mother stated that the child generally reacted to examinations with a rapidly increasing heart rate. Two non-orally fed children (three examinations) spat out the offered bolus and refused oral feeding. In one examination, the child cooperated without any problems. During thirteen FEES the children could be calmed down after a bit of fussing. In four cases, crying occurred throughout the examination. In all examinations, the parents stated that the children were generally doing well.

**Fig. 3:**
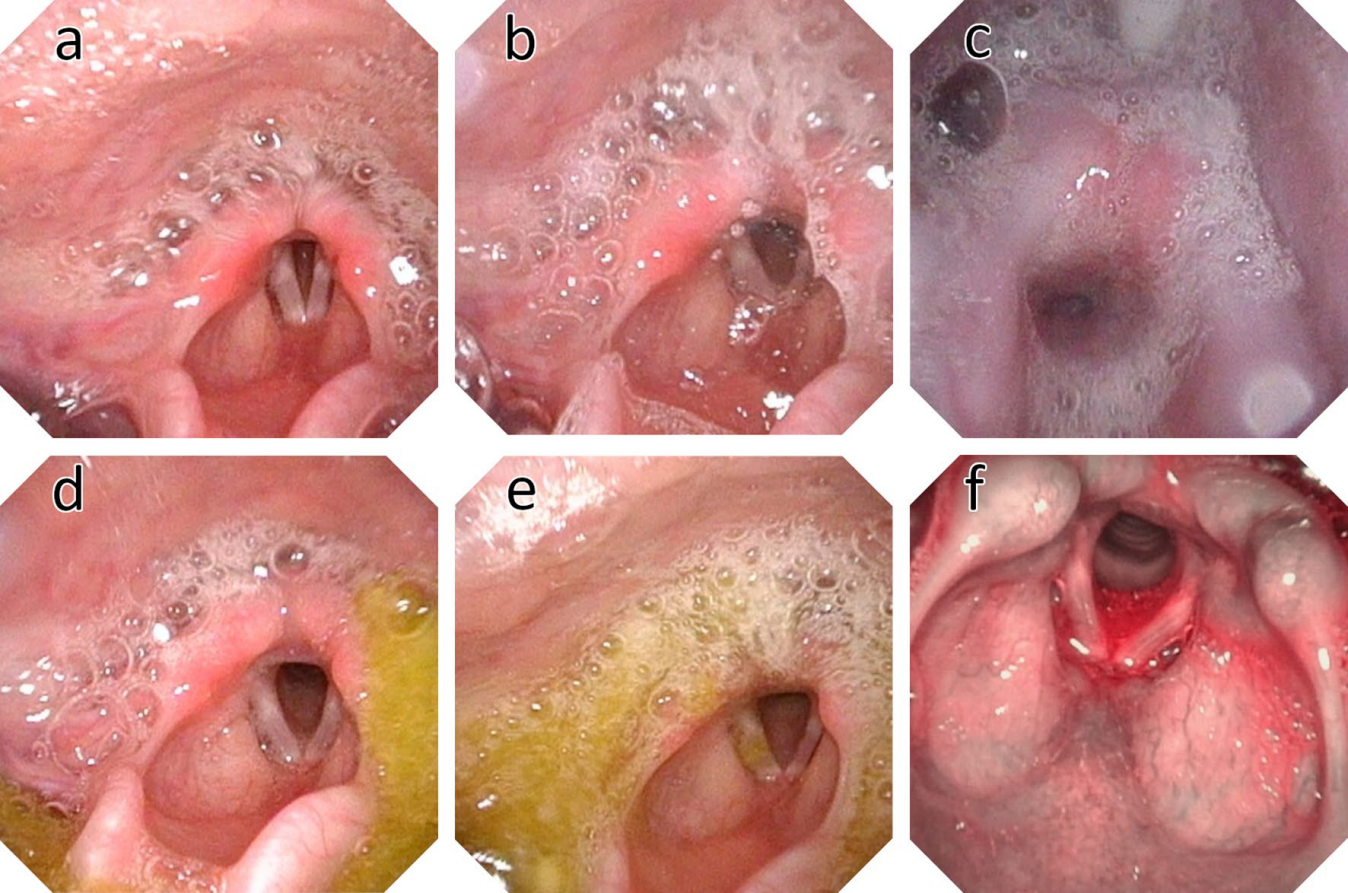
Examples from the sample: Murray Secretion Scale 1 (**a**), 2 (**b**) and 3 (**c**); spillage (**d**), penetration (**e**, see right vocal fold), and aspiration of dyed apple sauce (**f**). The added green food dye appears red under NBI (©Zang, 2022)

**Fig. 4:**
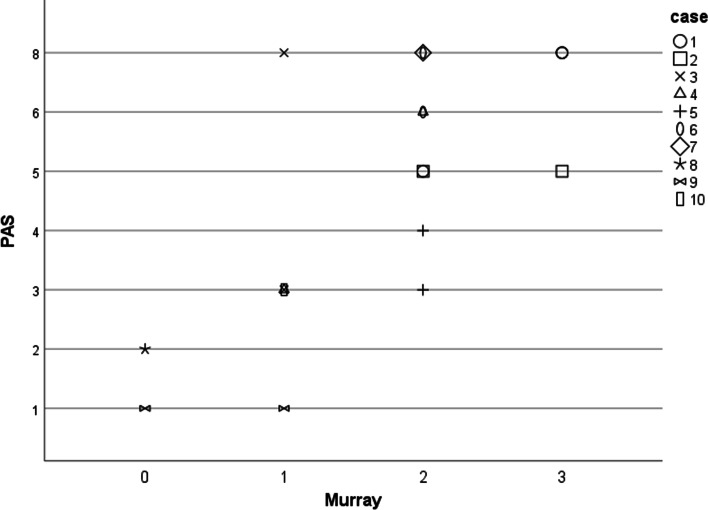
Scatterplot showing the association between Murray Secretion Scale and PAS

No anatomical pathologies in the pharynx or larynx were seen in the sample. Murray's Secretion Scale could be recorded in all FEES. Pharyngeal secretion pooling could be seen in 16 examinations in nine children. A foamy to white secretion was noticeable in most cases, which occasionally "bubbled up" from the trachea (Fig. 3). The scatterplot (Fig. 4) shows graphically that a higher Murray score is associated with a higher PAS.

A delay in initiating the swallowing reflex concerned all but one child in the first FEES and all children at follow-up. It is striking that four children had already completely lost their ability to swallow when they were included. Two of them started swallowing with a strong delay in the follow-up.

Due to a lack of bolus transport to the pharynx, spillage, PAS, and YPRSRS could not be scored in four FEES from two children. They were exclusively tube-fed, kept the bolus in the anterior oral cavity, and spat it out. PAS was scored for saliva in this case, and spillage and YPRSRS could not be captured. In summary, spillage occurred in six of the eight children who could swallow a bolus.

No penetration or aspiration was seen in one child at both evaluation points (PAS = 1). At the first examination, penetration alone (PAS 2–5) was seen in four children, and aspiration in four (PAS 6–8), including three silent ones (PAS = 8). At follow-up, penetration was seen in five children and aspiration in two, including one silent aspiration. Examples from the sample are given in Fig. 3.

Throughout all but one examination, post-swallow residues were seen. They ranged from trace to severe in the valleculae and the piriform sinus, regardless of the consistency of the bolus.

Laryngeal sensitivity was only determined in ten examinations of seven children. No reaction could be provoked in one child, weak response in five children, and physiological response in one. There were no changes in the laryngeal sensitivity at the follow-up.

### Association between clinical scores and FEES

The mixed linear model included a total of 18 examinations with FEES in ten children. None of the clinical scores were significantly associated with PAS (Table 3). These results must be interpreted with caution, since the PAS for penetration and aspiration of saliva was recorded in four examinations.

**Table 3:** Association between clinical scores and PAS

Parameter	Estimate^a^	95% CI^b^	*p* value
CHOP-INTEND	0.06	− 0.04 to 0.16	0.214
NdSSS	− 0.58	− 1.32 to 0.16	0.155
OrSAT	0.18	− 0.52 to 0.88	0.586

Dependent variable = PAS, random intercept = Case ID

^a^Estimate = regression coefficient

^b^95% CI = confidence interval of the regression coefficient

## Discussion

In this pilot study, we demonstrated that FEES is a feasible and safe instrumental procedure for infants and children with type 1 SMA when performed by experienced examiners. To some extent, all children were affected by dysphagia, and none of the currently available clinical scores provided reliable evidence of dysphagia measured by FEES. The applied protocol represents a valuable basis for future studies.

All planned FEES could be carried out well in terms of safety and feasibility. In severely affected children, some unique features, such as a short procedure or endoscopy in a lying position, could be easily implemented and were well tolerated by the children and their parents. Nevertheless, a particularly gentle approach is always indicated in this vulnerable group.

FEES' common findings were poor secretion management, delayed or absent swallowing reflex, spillage, penetration, aspiration, post-swallow residue, and reduced laryngeal sensitivity. Other studies performing VFSS found silent aspiration in four of five [2] and five of six children [3], respectively. In our study, silent aspiration was far less common than in these studies (3/9; 1/8). A comparison of the results is tricky, though. On the one hand, the data collected with FEES, such as secretion management, cannot be collected in VFSS; on the other hand, some implementation details from the studies, such as the bolus sizes offered, are unknown or vary. We only offered small amounts to non-oral feeders and avoided large volumes. Large volumes are more likely to cause aspiration; due to the respiratory impairment associated with the underlying disease, we deliberately did not perform this type of examination in this vulnerable group. Overall, there is a high risk of unsafe swallowing in this cohort.

It is noticeable that delayed swallowing (including the inability to swallow) occurred in all children. It probably resulted from decreasing muscular strength, including decreased larynx elevation. This observation also fits the clinically described symptoms from other studies [2, 21].

The assessment of pharyngeal secretion pooling (Murray Secretion Scale), a unique issue in this group, was a possible predictor for PAS. In exceptional cases when oral food intake is refused or an oral bolus cannot be administered, Murray Secretion Scale should be scored, and PAS for saliva should also be recorded. This study could not demonstrate a clear association between clinical scores and PAS, a problem that shows up generally [37]. An explanation could be that both NdSSS and OrSAT give an overall impression of the nutritional status and do not contain items assessed directly by a specialist. Future instruments intended to record the swallowing status validly must include a direct examination of the child in addition to anamnestic items. Until then, an instrumental examination of swallowing is indispensable and should be carried out early.

The approval studies of new pharmaceutical treatments for SMA mainly focus on muscle strength, motor function scales, and the need for ventilation support [14–16]. Given the success of different treatment options and the more prolonged survival of SMA patients, bulbar function and the potentially different efficacy of each treatment are becoming increasingly important. One can cautiously suspect that despite improvement in motor function, comparable improvement in swallowing function may not be achieved, as seen in other studies [15–17].

Several reasons can be assumed: (i) children who already lost their ability to swallow have not regained this ability or showed stagnation in the usually rapidly progressing swallowing development, (ii) the previous FEES rating scales and assessment instruments for swallowing development are not particularly sensitive to changes and show floor and ceiling effects, (iii) bulbar symptoms respond differently in comparison to motor function in the approved treatments, (iv) follow-up time was too short to record relevant changes. Overall, it is essential to note that even when motor development is satisfactory, dysphagia is a severe problem in children with SMA type 1 that requires careful and close monitoring.

### Limitations and considerations for future studies

The sample consisted of severely symptomatic children who were seen at varying ages. Serious adverse events related to the disease and the COVID-19 pandemic made data collection more complex. An evident weakness of the study is that no systematic pre–post and follow-up measurements could be carried out.

It must be pointed out that the assessment of PAS only for saliva in two children can undoubtedly be viewed critically. However, due to the rarity of the disease, consideration of each case is precious. Based on our experience, we believe these children would have received an even poorer PAS score for a food bolus. That should be considered for further interpretation of the data.

As a result of the recently started SMA newborn screening, a modified study design is required, and the children will be seen much earlier. This new study design will be implemented as part of the DySMA study (Dysphagia in Children with Spinal Muscular Atrophy). Therefore, clinical instruments are urgently needed to depict swallowing development sensitively and validly predict dysphagia as it progresses. There is no evidence that the swallowing problems can be halted entirely by treatment, even if treated at an early disease stage. In the future, FEES will be carried out regularly as part of routine diagnostics to monitor swallowing function in the context of new therapies. A multi-center approach and international collaboration will be essential for developing an overall protocol for clinical assessment combined with instrumental measurements.

## Conclusions

Due to the excellent representation of secretion management, no radiation exposure, the possibility to perform while breastfeeding, and the easy availability in contrast to VFSS, FEES is a suitable instrument to record dysphagia in children with type 1 SMA. The FEES protocol described provides valuable information about the unique features. The few clinical tools already used in children with type 1 SMA [3, 9, 21] offer an initial basis, but in their current form, they are not suitable for future studies to make an adequate statement on swallowing status and development. Systematic test development and focus studies on this topic are urgently needed.

## Data Availability

Data are tabulated in the manuscript. Further data are available from the corresponding author on reasonable request.

## References

[1] McGrattan KE, Graham RJ, DiDonato CJ, Darras BT (2021). Dysphagia phenotypes in spinal muscular atrophy: the past, present, and promise for the future. Am J Speech Lang Pathol.

[2] van der Heul AMB, Cuppen I, Wadman RI, Asselman F, Schoenmakers M, van de Woude DR, Gerrits E, van der Pol WL, van den Engel-Hoek L (2020). Feeding and swallowing problems in infants with spinal muscular atrophy type 1: an observational study. J Neuromuscul Dis.

[3] Choi YA, Suh DI, Chae JH, Shin HI (2020). Trajectory of change in the swallowing status in spinal muscular atrophy type I. Int J Pediatr Otorhinolaryngol.

[4] Davis RH, Godshall BJ, Seffrood E, Marcus M, LaSalle BA, Wong B, Schroth MK, Swoboda KJ (2014). Nutritional practices at a glance: spinal muscular atrophy type I nutrition survey findings. J Child Neurol.

[5] Pechmann A, Konig K, Bernert G, Schachtrup K, Schara U, Schorling D, Schwersenz I, Stein S, Tassoni A, Vogt S, Walter MC, Lochmuller H, Kirschner J (2019). SMArtCARE - A platform to collect real-life outcome data of patients with spinal muscular atrophy. Orphanet J Rare Dis.

[6] Bernal S, Alias L, Barcelo MJ, Also-Rallo E, Martinez-Hernandez R, Gamez J, Guillen-Navarro E, Rosell J, Hernando I, Rodriguez-Alvarez FJ, Borrego S, Millan JM, Hernandez-Chico C, Baiget M, Fuentes-Prior P, Tizzano EF (2010). The c.859G>C variant in the SMN2 gene is associated with types II and III SMA and originates from a common ancestor. J Med Genet.

[7] Ruhno C, McGovern VL, Avenarius MR, Snyder PJ, Prior TW, Nery FC, Muhtaseb A, Roggenbuck JS, Kissel JT, Sansone VA, Siranosian JJ, Johnstone AJ, Nwe PH, Zhang RZ, Swoboda KJ, Burghes AHM (2019). Complete sequencing of the SMN2 gene in SMA patients detects SMN gene deletion junctions and variants in SMN2 that modify the SMA phenotype. Hum Genet.

[8] Finkel RS, McDermott MP, Kaufmann P, Darras BT, Chung WK, Sproule DM, Kang PB, Foley AR, Yang ML, Martens WB, Oskoui M, Glanzman AM, Flickinger J, Montes J, Dunaway S, O'Hagen J, Quigley J, Riley S, Benton M, Ryan PA, Montgomery M, Marra J, Gooch C, De Vivo DC (2014). Observational study of spinal muscular atrophy type I and implications for clinical trials. Neurology.

[9] Weststrate H, Stimpson G, Thomas L, Scoto M, Johnson E, Stewart A, Muntoni F, Baranello G, Conway E, Group* SMAp-FW (2022). Evolution of bulbar function in spinal muscular atrophy type 1 treated with nusinersen. Dev Med Child Neurol.

[10] Pechmann A, Langer T, Schorling D, Stein S, Vogt S, Schara U, Kolbel H, Schwartz O, Hahn A, Giese K, Johannsen J, Denecke J, Weiss C, Theophil M, Kirschner J (2018). Evaluation of children with SMA type 1 under treatment with nusinersen within the expanded access program in Germany. J Neuromuscul Dis.

[11] Johannsen J, Weiss D, Schlenker F, Groth M, Denecke J (2021). Intrathecal administration of nusinersen in pediatric SMA patients with and without spine deformities: experiences and challenges over 3 years in a single center. Neuropediatrics.

[12] Passini MA, Bu J, Richards AM, Kinnecom C, Sardi SP, Stanek LM, Hua Y, Rigo F, Matson J, Hung G, Kaye EM, Shihabuddin LS, Krainer AR, Bennett CF, Cheng SH (2011). Antisense oligonucleotides delivered to the mouse CNS ameliorate symptoms of severe spinal muscular atrophy. Sci Transl Med.

[13] Hoy SM (2019). Onasemnogene abeparvovec: first global approval. Drugs.

[14] Baranello G, Darras BT, Day JW, Deconinck N, Klein A, Masson R, Mercuri E, Rose K, El-Khairi M, Gerber M, Gorni K, Khwaja O, Kletzl H, Scalco RS, Seabrook T, Fontoura P, Servais L, Group FW (2021). Risdiplam in type 1 spinal muscular atrophy. N Engl J Med.

[15] Day JW, Finkel RS, Chiriboga CA, Connolly AM, Crawford TO, Darras BT, Iannaccone ST, Kuntz NL, Pena LDM, Shieh PB, Smith EC, Kwon JM, Zaidman CM, Schultz M, Feltner DE, Tauscher-Wisniewski S, Ouyang H, Chand DH, Sproule DM, Macek TA, Mendell JR (2021). Onasemnogene abeparvovec gene therapy for symptomatic infantile-onset spinal muscular atrophy in patients with two copies of SMN2 (STR1VE): an open-label, single-arm, multicentre, phase 3 trial. Lancet Neurol.

[16] Finkel RS, Mercuri E, Darras BT, Connolly AM, Kuntz NL, Kirschner J, Chiriboga CA, Saito K, Servais L, Tizzano E, Topaloglu H, Tulinius M, Montes J, Glanzman AM, Bishop K, Zhong ZJ, Gheuens S, Bennett CF, Schneider E, Farwell W, De Vivo DC, Group ES (2017). Nusinersen versus Sham Control in Infantile-Onset Spinal Muscular Atrophy. N Engl J Med.

[17] Weiss C, Ziegler A, Becker LL, Johannsen J, Brennenstuhl H, Schreiber G, Flotats-Bastardas M, Stoltenburg C, Hartmann H, Illsinger S, Denecke J, Pechmann A, Muller-Felber W, Vill K, Blaschek A, Smitka M, van der Stam L, Weiss K, Winter B, Goldhahn K, Plecko B, Horber V, Bernert G, Husain RA, Rauscher C, Trollmann R, Garbade SF, Hahn A, von der Hagen M, Kaindl AM (2021). Gene replacement therapy with onasemnogene abeparvovec in children with spinal muscular atrophy aged 24 months or younger and bodyweight up to 15 kg: an observational cohort study. Lancet Child Adolesc Health.

[18] Mendell JR, Al-Zaidy S, Shell R, Arnold WD, Rodino-Klapac LR, Prior TW, Lowes L, Alfano L, Berry K, Church K, Kissel JT, Nagendran S, L'Italien J, Sproule DM, Wells C, Cardenas JA, Heitzer MD, Kaspar A, Corcoran S, Braun L, Likhite S, Miranda C, Meyer K, Foust KD, Burghes AHM, Kaspar BK (2017). Single-dose gene-replacement therapy for spinal muscular atrophy. N Engl J Med.

[19] Waldrop MA, Karingada C, Storey MA, Powers B, Iammarino MA, Miller NF, Alfano LN, Noritz G, Rossman I, Ginsberg M, Mosher KA, Broomall E, Goldstein J, Bass N, Lowes LP, Tsao CY, Mendell JR, Connolly AM (2020). Gene therapy for spinal muscular atrophy: safety and early outcomes. Pediatrics.

[20] Wada A, Kawakami M, Liu M, Otaka E, Nishimura A, Liu F, Otsuka T (2015). Development of a new scale for dysphagia in patients with progressive neuromuscular diseases: the Neuromuscular Disease Swallowing Status Scale (NdSSS). J Neurol.

[21] Berti B, Fanelli L, de Sanctis R, Onesimo R, Palermo C, Leone D, Carnicella S, Norcia G, Forcina N, Coratti G, Giorgio V, Cerchiari A, Lucibello S, Finkel R, Pane M, Mercuri E (2021). Oral and swallowing abilities tool (OrSAT) for type 1 SMA patients: development of a new module. J Neuromuscul Dis.

[22] Crary MA, Mann GDC, Groher ME (2005). Initial psychometric assessment of a functional oral intake scale for dysphagia in stroke patients. Arch Phys Med Rehab.

[23] Durkin ET, Schroth MK, Helin M, Shaaban AF (2008). Early laparoscopic fundoplication and gastrostomy in infants with spinal muscular atrophy type I. J Pediatr Surg.

[24] Rosenbek JC, Robbins JA, Roecker EB, Coyle JL, Wood JL (1996). A penetration-aspiration scale. Dysphagia.

[25] Glanzman AM, Mazzone E, Main M, Pelliccioni M, Wood J, Swoboda KJ, Scott C, Pane M, Messina S, Bertini E, Mercuri E, Finkel RS (2010). The Children's Hospital of Philadelphia Infant Test of Neuromuscular Disorders (CHOP INTEND): test development and reliability. Neuromuscul Disord.

[26] Zang J, Nienstedt JC, Koseki JC, Niessen A, Flugel T, Kim SH, Pflug C (2021). Pediatric flexible endoscopic evaluation of swallowing: critical analysis of implementation and future perspectives. Dysphagia.

[27] Zang J, Kiehn S, Flugel T, Koseki JC, Niessen A, Kim SH, Pflug C, Nienstedt JC (2022). Implementation of pediatric flexible-endoscopic evaluation of swallowing: a systematic review and recommendations for future research. Dysphagia.

[28] Langmore SE, Schatz K, Olsen N (1988). Fiberoptic endoscopic examination of swallowing safety: a new procedure. Dysphagia.

[29] Miller CK, Schroeder JW, Langmore S (2020). Fiberoptic endoscopic evaluation of swallowing across the age spectrum. Am J Speech Lang Pathol.

[30] Murray J, Langmore SE, Ginsberg S, Dostie A (1996). The significance of accumulated oropharyngeal secretions and swallowing frequency in predicting aspiration. Dysphagia.

[31] Warnecke T, Oelenberg S, Teismann I, Hamacher C, Lohmann H, Ringelstein EB, Dziewas R (2010). Endoscopic characteristics and levodopa responsiveness of swallowing function in progressive supranuclear palsy. Mov Disord.

[32] Miller CK, Willging JP (2020). Fiberoptic endoscopic evaluation of swallowing in infants and children: protocol, safety, and clinical efficacy: 25 years of experience. Ann Otol Rhinol Laryngol.

[33] Langmore SE, Olney RK, Lomen-Hoerth C, Miller BL (2007). Dysphagia in patients with frontotemporal lobar dementia. Arch Neurol.

[34] Neubauer PD, Rademaker AW, Leder SB (2015). The Yale Pharyngeal Residue Severity Rating Scale: an anatomically defined and image-based tool. Dysphagia.

[35] Nienstedt JC, Muller F, Niessen A, Fleischer S, Koseki JC, Flugel T, Pflug C (2017). Narrow band imaging enhances the detection rate of penetration and aspiration in FEES. Dysphagia.

[36] Marian T, Schroder JB, Muhle P, Claus I, Riecker A, Warnecke T, Suntrup-Krueger S, Dziewas R (2017). Pharyngolaryngeal sensory deficits in patients with middle cerebral artery infarction: lateralization and relation to overall dysphagia severity. Cerebrovasc Dis Extra.

[37] Garand KLF, McCullough G, Crary M, Arvedson JC, Dodrill P (2020). Assessment across the life span: the clinical swallow evaluation. Am J Speech Lang Pathol.

